# Transcriptomics of Human Brain Tissue in Parkinson’s Disease: a Comparison of Bulk and Single-cell RNA Sequencing

**DOI:** 10.1007/s12035-024-04124-5

**Published:** 2024-04-05

**Authors:** Michael R. Fiorini, Allison A. Dilliott, Rhalena A. Thomas, Sali M. K. Farhan

**Affiliations:** 1 The Montreal Neurological Institute-Hospital, Montreal, QC Canada; 2 Department of Human Genetics, McGill University, Montreal, QC Canada; 3 Department of Neurology and Neurosurgery, McGill University, Montreal, QC Canada

**Keywords:** RNA sequencing, Single cell, Single nucleus, Transcriptome, Parkinson’s disease, Neurodegenerative disease

## Abstract

Parkinson’s disease (PD) is a chronic and progressive neurodegenerative disease leading to motor dysfunction and, in some cases, dementia. Transcriptome analysis is one promising approach for characterizing PD and other neurodegenerative disorders by informing how specific disease events influence gene expression and contribute to pathogenesis. With the emergence of single-cell and single-nucleus RNA sequencing (scnRNA-seq) technologies, the transcriptional landscape of neurodegenerative diseases can now be described at the cellular level. As the application of scnRNA-seq is becoming routine, it calls to question how results at a single-cell resolution compare to those obtained from RNA sequencing of whole tissues (bulk RNA-seq), whether the findings are compatible, and how the assays are complimentary for unraveling the elusive transcriptional changes that drive neurodegenerative disease. Herein, we review the studies that have leveraged RNA-seq technologies to investigate PD. Through the integration of bulk and scnRNA-seq findings from human, post-mortem brain tissue, we use the PD literature as a case study to evaluate the compatibility of the results generated from each assay and demonstrate the complementarity of the sequencing technologies. Finally, through the lens of the PD transcriptomic literature, we evaluate the current feasibility of bulk and scnRNA-seq technologies to illustrate the necessity of both technologies for achieving a comprehensive insight into the mechanism by which gene expression promotes neurodegenerative disease. We conclude that the continued application of both assays will provide the greatest insight into neurodegenerative disease pathology, providing both cell-specific and whole-tissue level information.

## Background

Parkinson’s disease (PD) is the second most common neurodegenerative disease, presenting with motor system disfunction including slow movements, tremor, gait and balance disturbances, as well as non-motor system symptoms including loss of smell, cognitive decline, and dementia [1]. PD affects approximately 1% of the population above the age of 60 [2] and displays heritability estimates of approximately 30% [3, 4]. Individuals carrying known PD-associated genetic variants can display a wide range of disease presentations including variable age of onset, symptoms, and progression rates. In addition to genetic risk factors, specific environmental exposures such as organochlorine pesticides, including rotenone and paraquat [5], cocaine and amphetamines [6], and 1-methyl-4-phenyl-1,2,3,6-tetrahydropyridine [7], have been identified as major environmental contributors to parkinsonism.

The presence of Lewy bodies and the progressive loss of dopaminergic neurons (DaN) in the substantia nigra pars compacta (SNpc) are the main pathological features of PD [8, 9]. Additionally, multiple biological pathways encompassing mitochondrial dysfunction, neuroinflammation, and protein turnover have been implicated in the disease process and are believed to intersect with the accumulation of α-synuclein—the primary component of Lewy bodies—and the elevated degeneration susceptibility of DaNs in the SNpc. While the discovery of the loss of DaNs in PD has led to attempted treatments such as dopamine substitution and deep brain stimulation, the disease remains incurable, as available therapeutics can only target specific symptoms or minimally slow disease progression [10]. Indeed, elucidating the complex interactions between perturbed pathways and the neuroanatomical changes seen in the parkinsonian brain will advance the development of therapeutics in PD.

One promising approach for characterizing pathological interactions in PD is transcriptome analysis, which allows for global differential gene expression profiling. By comparing samples from individuals with disease to samples from healthy controls, we can learn how specific disease events influence the dynamic nature of gene expression or perhaps how these events lead to pathogenesis [11]. After identifying statistically significant differentially expressed genes or transcripts, we can apply gene set enrichment analysis (GSEA) to learn whether the transcriptional products are implicated in common biological pathways. Understanding how these pathways become disrupted, whether via hypo- or hyper-activity, can guide us in implementing the appropriate therapeutic strategy for halting disease progression [12].

In the early 1990s, the ability to interrogate RNA sequences on a large scale was facilitated by the sequential introductions of the expressed sequence tag (EST) and the serial analysis of gene expression (SAGE) methods [13]. However, microarray (hybridization-based) transcriptome profiling emerged in 1995 and superseded EST and SAGE given its rapidity and affordability, allowing researchers to employ large-scale studies to generate quantitative gene expression changes in disease states [14]. Despite advancements, microarray-based expression analyses are dependent on cross-hybridization, and their potential is restricted by a requirement for a priori knowledge of genetic sequences and a limited capacity to detect and quantify low- and high-abundance transcripts [15]. Moreover, sub-optimally designed probes and incorrect probe annotations reduce reproducibility and cross-platform consistency [16].

**Fig. 1:**
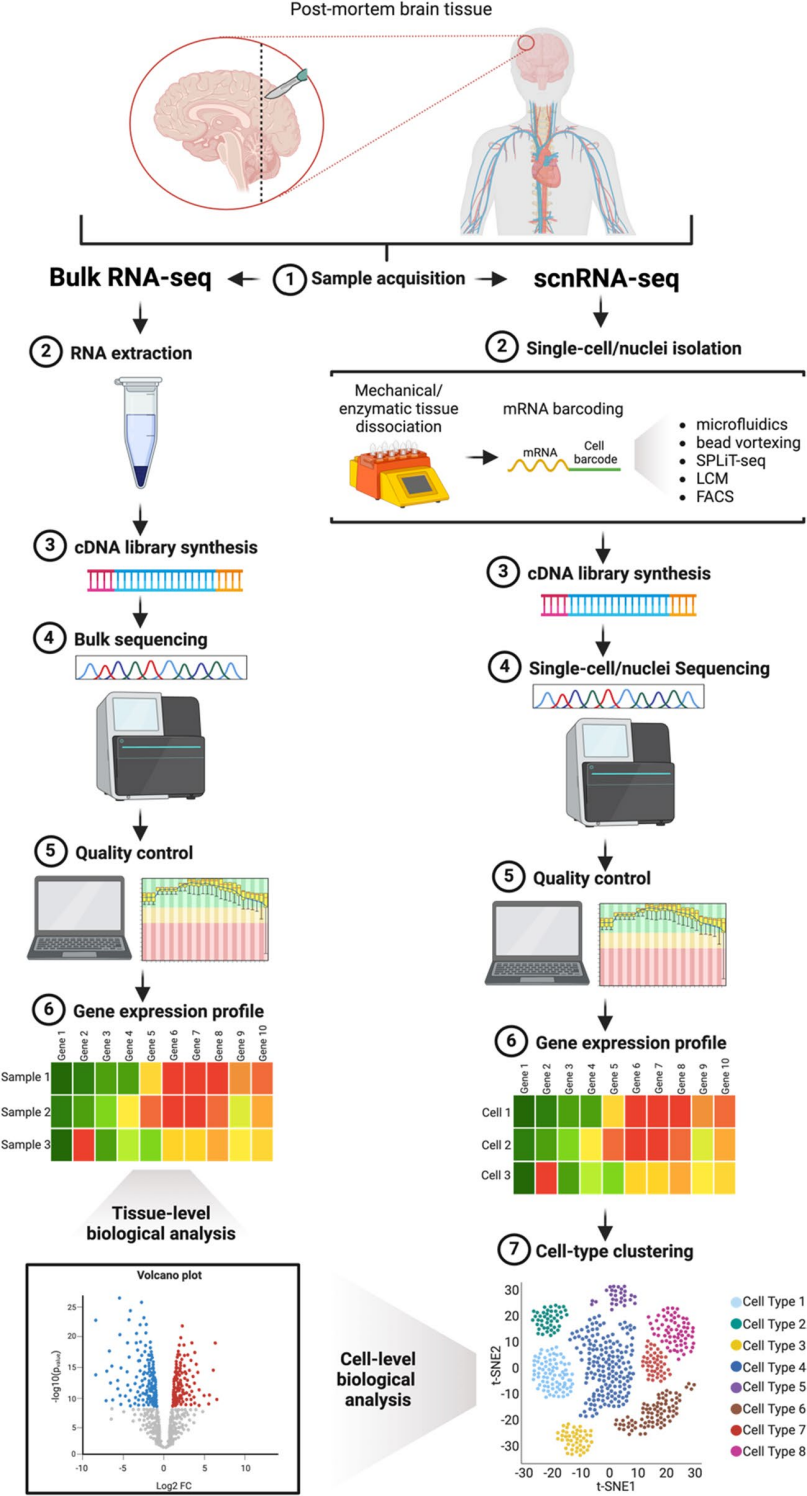
Bulk and single-cell/single-nucleus RNA sequencing workflows. Samples are acquired from disease-relevant post-mortem tissue. Single-cell/nuclei isolation in the single-cell and single-nucleus RNA sequencing (scnRNA-seq) workflow is achieved by first dissociating the tissue, followed by mRNA barcoding via microfluidics, bead vortexing, SPLiT-seq, laser capture microdissection (LCM), or fluorescence activated cell sorting (FACS) to tag the transcripts from each cell with cell-specific barcodes. In bulk RNA-seq, RNA must be extracted from the cells or tissue to be analyzed prior to library preparation. Complementary DNA (cDNA) library synthesis allows RNA-seq experiments to be carried out on technically mature commercial instruments designed for DNA-based sequencing. Sequencing on next-generation sequencing platforms provides “reads,” defined as strings of sequence data. Quality control of sequencing data ensures the absence of intrinsic biases associated with library preparation, including nucleotide composition bias and GC bias. The number of reads mapped to each gene is used to generate gene expression profiles for each sample or cell for bulk and scnRNA-seq, respectively. Computational cell-type clustering in the scnRNA-seq workflow groups cells with similar expression profiles to identify distinct cellular species. The resulting expression data from bulk RNA-seq and scnRNA-seq can be used for tissue-level or cell-level biological analyses, respectively

Next-generation RNA sequencing (RNA-seq) is a more efficient, less-expensive technology capable of transcriptome-wide analyses [11]. RNA-seq approaches begin with a cell suspension comprised of dissociated cells from the tissue of interest. Entire RNA molecules are used to generate complementary DNA (cDNA) sequences, which are fragmented and sequenced by massively parallel deep sequencing. The sequences are then aligned to a reference genome to produce a tissue-wide gene expression profile (Fig. 1). Referred to as total or bulk RNA-seq, this method involves RNA being extracted from all cells within a tissue (typically blood, skin, or muscle) and mixed together, resulting in an average expressional profile [17]. However, in the absence of single-cell isolation, an inherent limitation of bulk RNA-seq stems from sequencing tissues comprised of many cell types. As such, biological interpretations are impeded by the lack of cell-type specific gene expression.

Single-cell RNA-seq (scRNA-seq) was introduced in 2009 by Tang et al. to capture an entire transcriptome library of one cell [18]. Further, in 2011, Islam et al. applied scRNA-seq to highly multiplexed samples comprising 96 cells to generate large-scale single-cell expression profiles [19]. In 2015, Drop-seq introduced the first scalable method to obtain single-cell transcriptomes on thousands of cells by separating individual cells into aqueous droplets, tagging each cell’s RNA with a unique molecular barcode, and sequencing all cells simultaneously [20]. In addition to microfluidic technologies (such as Drop-seq), bead vortexing, in situ barcoding (SPLiT-seq), laser capture microdissection (LCM), and fluorescent activated cell sorting (FACS) can also be used to isolate single cells prior to sequencing. By sequencing individual cells, scRNA-seq mitigates the limitations of bulk RNA-seq by providing transcriptional data at a single-cell resolution, capturing cell-to-cell variability in gene expression, and revealing the cell-type heterogeneity of a tissue. Accordingly, scRNA-seq is particularly important for studying neurodegenerative disease as it facilitates the exploration of specific cell types that are most vulnerable to degeneration and can inform mechanisms by which cell populations interact to promote disease pathology.

One limitation of scRNA-seq is that it may provide a biased representation of captured cell types, as certain cellular species are more vulnerable to the dissociation process [21]. For example, neuronal cells from the human neocortex dissociate poorly compared to non-neuronal cell types and are therefore underrepresented in cell suspensions [22]. One way to overcome this limitation is to harness single-nucleus RNA-seq (snRNA-seq), which is similar to scRNA-seq as it is used to sequence transcriptomes of individual cells; however, only nuclear RNA is captured. Given that nuclei are more resistant to mechanical stress, snRNA-seq ensures that vulnerable cellular species are adequately represented in the final dataset, and, unlike single cells, they can be isolated from frozen post-mortem tissues [23–25]. Furthermore, it has been shown that nuclei yield expression profiles resembling entire cells, thus making snRNA-seq an adequate substitution for scRNA-seq in most applications [24].

scRNA-seq and snRNA-seq (scnRNA-seq) workflows resemble bulk RNA-seq; however, single cells or nuclei must be isolated by microfluidics, bead vortexing, in situ barcoding*,* FACS, or LCM prior to cDNA synthesis and sequencing. After cells or nuclei have been sequenced, the results are embedded onto gene expression space and computationally clustered to identify transcriptionally unique populations that can be annotated based on the expression of established marker genes, enabling cell-type specific analyses downstream (Fig. 1). Importantly, the current application of scnRNA-seq assays to cohort-scale analyses is restricted by relatively high monetary cost and stringent single-cell isolation processes.

In this review, we leverage the transcriptional landscape of the Parkinsonian brain, which has been thoroughly investigated using both bulk and single-cell approaches, as a case study for evaluating the utility of bulk and scnRNA-seq in neurodegeneration. By comparing and integrating the findings derived from short-read next-generation sequencing methods, we evaluate whether transcriptomic analyses at different resolutions provide compatible results and demonstrate the complementarity of both assays. Finally, we evaluate the current feasibility of bulk and scnRNA-seq methods through the lens of the PD transcriptomic literature to illustrate the necessity of both technologies for achieving a holistic insight into the mechanism by which gene expression promotes neurodegenerative disease.

**Fig. 2:**
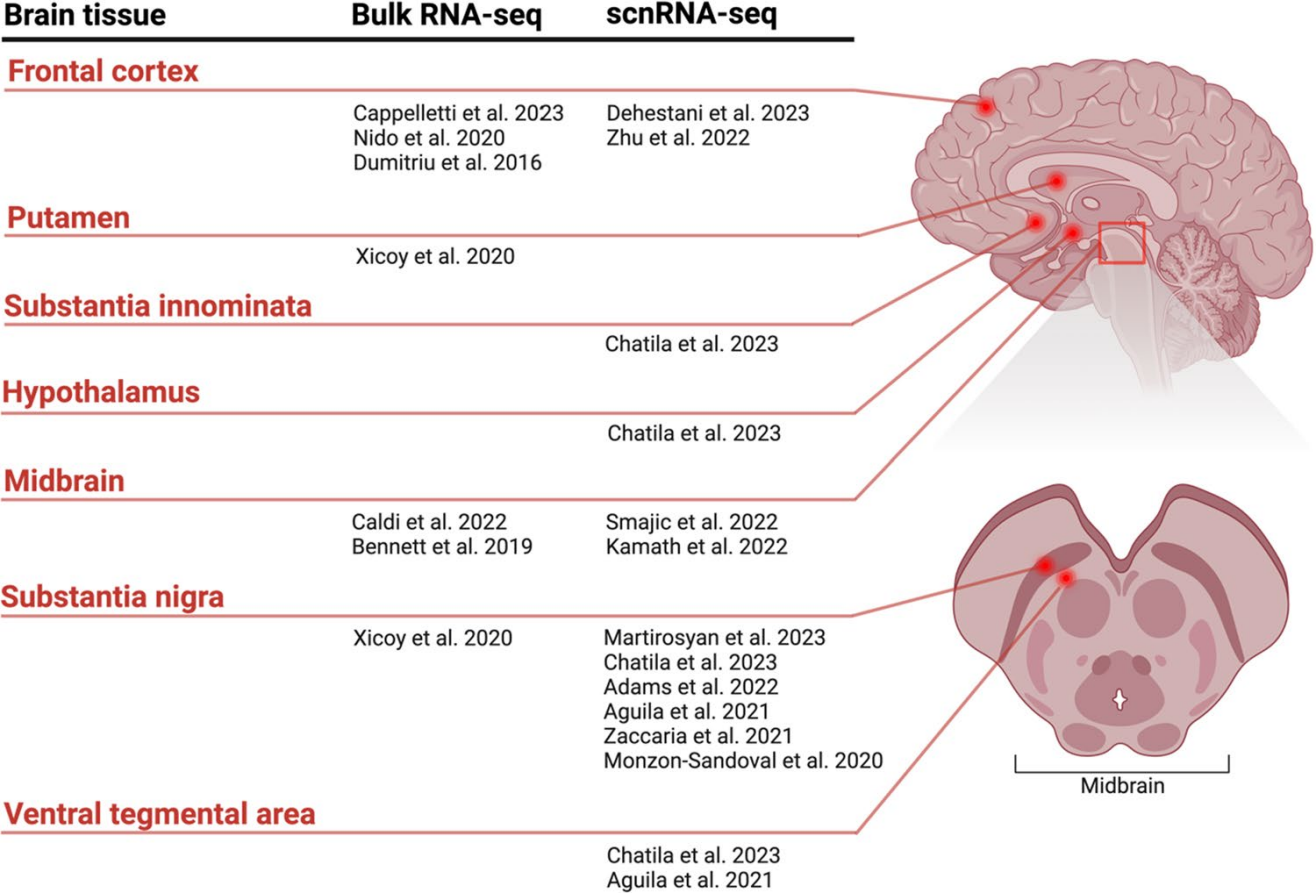
RNA sequencing (RNA-seq) studies on Parkinson’s disease (PD). RNA-seq studies performed on human post-mortem brain tissue to study PD, which were reviewed herein. Studies are categorized by RNA-seq technology, and brain tissue. Abbreviations: scnRNA-seq, single-cell and single-nucleus RNA sequencing

Herein, we compare and integrate findings from bulk and scnRNA-seq studies that have explored the post-mortem frontal cortex, prefrontal cortex, midbrain, substantia nigra (SN), ventral tegmental area (VTA), substantia innominata, and hypothalamus in the context of PD (Fig. 2). We focus on the loss of DaNs in the SNpc and highlight key pathways that overlap across RNA-seq studies including synaptic dysfunction, inflammation, mitochondrial dysfunction, and the unfolded protein response (UPR). A comprehensive analysis of these RNA-seq studies is shown in Table 1. To the best of our knowledge, we have included all RNA-seq analyses of human post-mortem brain tissue focussed on PD.

**Table 1:** Comprehensive description of RNA sequencing (RNA-seq) studies on Parkinson’s disease (PD) human post-mortem brain tissue

Citation	PD subjects	Control subjects	Tissue	Sample aquisition	Computational analyses	Downregulated pathways in PD	Upregulated pathways in PD	Enriched pathways in PD
Single-cell/single-nucleus RNA-seq								
Dehestani et al. BioRxiv 2023	4	2	Prefrontal cortex	Post-mortem	• Cell-type association with PD risk genes • Trajectory analysis • WGCNA	Oligodendrocyte: • Protein targetting to to mitochondria • Regulation of potassium ion transport Oligodendrocyte precursor cells: • Modulation of chemical synaptic transmission • Cholesterol biosynthesis	Oligodendrocyte: • Negative regulation of inclusion body assembly • Regulation of micortubule nucleation Oligodendrocyte precursor cells: • Positive regulation of tau-protein kinase activity • Regulation of protein polymerization	Not described
Martirosyan et al. Molecular Neurodegeneration 2023	15	14	Substantia nigra	Post-mortem	• DEG analysis • GO enrichment analysis • KEGG enrichment analysis	Not described	Not described	Dopaminergic neurons: • Energy production • Cholesterol metabolism • Iron transport • Oxidative stress • Unfolded protein response GABAergic neurons: • Unfolded protein response • Oxidative stress • Energy production • Iron transport Astrocytes: • Synaptic function • Ion and calcium transport • Unfolded protein response Microglia: • Protein folding • Unfolded protein response • Immune response Oligodendrocytes: • Axon development • Synapse organization • Ion transport • Synaptic vessicle cycle • Mitochondrial function • Apoptosis • Immune response • Myelination
Chatila et al. BioRxiv 2023	19	14	• Substantia nigra • Ventral tegmental area • Substantia inominata • Hypothalamus	Post-mortem	• DEG analysis • GO enrichment analysis • Cell type abundance analysis • Enrichment of PD risk genes • ATAC-seq analysis	Microglia from substantia nigra: • Unfolded protein response • RNA splicing • Antigen presentation	Micorglia from substantia nigra: • Cell devision	Not described
Adams et al. MedRxiv 2022	14	17	Substantia nigra	Post-mortem	• DEG analysis • GO enrichment analysis • Cell-type cluster annotation • Single-cell chromatin profiles • Gene expression and chromatin accessibility profile analysis • Gene expression and ATAC peak relationship analysis • Cell-type specific pseudopathogenesis analysis • Functional subtype gene module analysis • Motif enrichment analysis • SNP-containing peaks analysis	Downregulated with advancing pseudopathogenesis in oligodendrocytes: • Myelination • Receptor clustering • Regulation of mebrane potential Downregulated with advancing pseudopathogenesis in microglia: • Cell adhesion • Chemotaxis • Neuronal support	Upregulated with advancing pseudopathogenesis in oligodendrocytes: • Response to unfolded protein • Chaperone-mediated autophagy • Negative regulation of cell death Upregulated with advancing pseudopathogenesis in microglia: • Immune activation • Cytokine-mediated signaling pathway • Apoptosis	Not described
Kamath et al. Nature Neuroscience 2022	10	9	Midbrain	Post-mortem	• DEG analysis • Cell type clustering and annotation • Identification of differentially regulated regulons • Integration of DaN across species • Differential abundance of cell types in PD • Cell-type specific heritability enrichment analysis • Familial PD variant enrichment analysis • GSEA of transcription factors across DaN subtypes	Not described	Not described	Not described
Smajic et al. Brain 2022	5	6	Midbrain	Post-mortem	• Differential cell-type composition • Glial cell trajectory reconstruction • Glial cell DEG analysis • Functional enrichment analysis • Association analysis of cell-type specific expressed genes with genetic risk of PD	Oligodendrocyte: • Neuronal maintaining pathways	Oligodendrocyte: • Response to unfolded protein pathways	Activated microglia: • Cytokine secretion • Stress response to unfolded protein Astrocytes: • Unfolded protein response
Zhu et al. BioRxiv 2022	6	6	Prefrontal cortex	Post-mortem	• DEG analysis • GO functinal enrichment analysis • RNA velocity analysis • Annotation of post-GWAS candidate genes • Differential protein expression analysis • WGCA on proteomic data	Excitatory neurons: • Protein folding • Unfolded protein response Inhibitory neurons: • Unfolded protein response • Receptor mediated endocytosis Microglia: • Phospholipid binding • Virus receptor activity Astrocytes: • Coagulation Brain-resident T cells: • GATOR2 complex • Receptor antagonist actvity • Enhancer binding	Neurons: • Axonogensis • Pre-synaptic assembly Oligodendrocytes: • Protein complex assembly • Cytoskeleton organization Astrocytes: • Detoxification of heavy metals Microglia: • Myeloid cell differentiation • Regulation of hemopoiesis • Glycolytic process Oligodendrocyte precursor cells: • Regulation of cell growth • Positive neurogenesis Brain-resident T cells: • Homeostatsis of cell number • Regulation of T cell differentiation	Not described
Aguila et al. Frontiers in Molecular Neuroscience 2021	Not applicable	18	• SNpc • VTA	Post-mortem LCM	• DEG analysis between SNpc and VTA DaN • Protein–protein interaction network analysis (DEGs between SNpc and VTA)	Not described	Not described	Not described
Zaccaria et al. Cellular and Molecular Neurobiology 2021	3	3	SNpc	Post-mortem LCM	• DEG analysis • Differential protein expression analysis	Not described	Not described	Not described
Monzon-Sandoval et al. Annals of Neurology 2020	Not applicable	7	SNpc	Post-mortem LCM	• DEG analysis • GO enrichment analysis • Co-expression analysis • Protein–protein interaction analysis • Phenotypic-linkage network analysis	Relative to dorsal neurons (higher expression in dorsal neurons): • Negative regulation of cell migration • Canonical Wnt signaling • Positive regulation of proteosomal ubiquitin dependent protein catabolic process	Relative to dorsal neurons (lower expression in dorsal neurons): • Receptor mediated endocytosis • Calcium ion homeostasis • Potassium ion transport • Regulation of cell proliferation • Cell surface receptor signalling • Central nervous system development • Cellular response to tumor necrosis factor • Insulin stimulus	Not described
Bulk RNA-seq								
Cappelletti et al. Acta Neuropathologica 2023	61	23	Frontal cortex	Post-mortem	• Cell type deconvolution • DEG analysis • Expression pattern analysis across disease stages • Functional enrichment analysis • Expression quantitative trait locus analyses	• ATP metabolic processes	• Immune response	• Mitochondrial-related processes • Synaptic signal release • Axonal maintenance
Caldi et al. Clinical and Translational Medicine 2022	25	14	Midbrain	Post-mortem	• DEG analysis • Differential protein expression analysis • Small RNA-seq intergrated to target transcripts identified by total RNA-seq • Gene-protein expression integration • GO enrichment analyses • KEGG pathway enrichment analyses • Cell-type deconvolution • WGCNA	• Mitochondrial-related processes	• Immune-inflammation • Response to stress/apoptosis • Metabolic/biosynthesis processes • Cytoskeleton organization	Not described
Nido et al. Acta Neuropathologica communications 2020	28	22	Prefrontal cortex	Post-mortem	• DEG analysis • Cell-type deconvolution • GO enrichment analysis	• Endoplasmic reticulum-related processes • Lipid oxidation	Protein folding	Not described
Xicoy et al. Cells 2020	19	19	• Substantia nigra • Putamen	Post-mortem	• DEG analysis • Lipid profiling • GO enrichment analysis	Substantia nigra: • Regulation of neurotransmitter levels • Signal release • Neurotransmitter transport Putamen: • Synapse organization • Cartilage development • Synapse assembly	Substantia nigra: • Response to heat • Chaperone-mediated protein folding	Substantia nigra: • Signal release • Neurotransmitter transport •Amine transport Putamen: • Synapse organization • Cartilage development • Prostaglandin transport
Bennett et al. Frontiers in Neuroscience 2019	12	8	Midbrain	Post-mortem	2-way ANOVA	Not applicable	Not applicable	Not applicable
Dumitriu et al. BMC Medical Genomics 2016	29	44	Prefrontal cortex	Post-mortem	• DEG analysis • Proteomics analysis • GO enrichment analysis • MSigDB enrichment analysis • Identification of genes under the reguatory control of PD-associated genome-wide significant single nucleotide polymorphisms	Not described	Not described	Transcriptomics: • Response to unfolded protein • COP9 signalosome • Unfolded protein binding • KEGG antigen processing and presentation • Reactome mediated NFKB activation via dai Proteomics: • Mitochondrial electron transport • NADH to ubiquinone • Mitochondrial respiratory chain complex 1 • NADH dehydrogenase activity • Reactome respiratory electron transport (MSigDB)

### Selective Vulnerability of DaNs in the SNpc

The progressive loss of DaNs in the SNpc is one of the main pathological features of PD [9]; 50% of DaNs were found to be lost through histological analysis of post-mortem tissue [26]. Interestingly, some DaNs in the SNpc appear resistant to degeneration and survive into the late stages of the disease, while DaNs in the VTA (adjacent to the substantia nigra) of PD patients show a much lower magnitude of degeneration compared to those in the SNpc [27–32]. Yet, the molecular basis leading to the vulnerability of a subset of DaNs in the SNpc has remained elusive.

The resolution of bulk RNA-seq is limited to providing a whole-tissue-level understanding of the transcriptome, permitting the comparison of SNpc to VTA but not subtypes within one region. One exciting application for scnRNA-seq assays in PD is the ability to characterize the transcriptional landscape of the distinct cellular subgroups of DaNs that are particularly vulnerable or resistant to degeneration within the SNpc. Transcriptional data at a single-cell resolution can then inform disease-related changes in cell-type proportions and the presence of transcriptionally distinct, disease-specific cell types. For example, upon selective enrichment of SNpc samples for neurons, snRNA-seq corroborated that DaNs from the SNpc showed the largest decline in PD samples compared to non-PD controls by identifying one highly vulnerable, transcriptionally distinct DaN subtype that was selectively depleted in PD samples and two transcriptionally distinct DaN subtypes that were selectively enriched in PD samples [33], implying that these subtypes are resistant to PD-related cell death. Consistent with patterns of DaN loss in PD [34], single-molecule fluorescence in situ hybridization (FISH) revealed that the highly vulnerable DaN subtype was confined to the ventral tier of the SNpc and that the two resistant DaN subtypes were confined to the dorsal tier [33]. Importantly, it should be noted that distinct cellular populations identified by scnRNA-seq warrant further validation using different modalities, such as FISH, histology, or spatial multiomics to support the findings.

Although the molecular basis responsible for the selective vulnerability of a subset of DaNs in the PD SNpc remains elusive, snRNA-seq of post-mortem midbrain tissues has provided notable insights. The vulnerable, ventrally located DaN subtype—selectively depleted in PD samples—from the SNpc were most strongly enriched for the expression of PD risk genes identified by genome-wide association studies (GWAS), including *SNCA*, *MAPT*, *GAK*, *WNT3*, and *IGSF9B* [33, 35], suggesting that the presence of these genetic risk factors, and possibly their altered activity in these vulnerable neurons, comprises an inherent risk that influences their survival in PD. These same DaNs were enriched for pathways related to regulation of neuron death and WNT signaling [33]. Similarly, in a separate study, a transcriptionally distinct neuronal subtype was predominantly found in the midbrain of PD samples compared to non-PD controls, which showed elevated expression of *CADPS2* and *TIAM1* [36]. *CADPS2* has been previously linked to catecholamine uptake, while *TIAM1* is involved in WNT/DVl/RAC1 signalling [37]. The WNT signalling pathway is critical for development and maintenance of DaNs [38]; thus, the convergence of both snRNA-seq analyses for aberrant WNT signalling in vulnerable DaNs emphasizes this pathway as an important contributor to the selective degeneration of neuronal cells in the PD midbrain. Indeed, there is evidence showing that the loss of function of parkin, a PD-associated protein, can lead to increased WNT signaling and consequent post-mitotic DaN death [39]. Despite the apparent importance of the WNT signaling pathway in the PD disease process, bulk RNA-seq analyses of post-mortem PD brains have yet to find evidence for this pathological feature, likely owing to the relative sparsity of DaNs that results in expressional signals being diminished upon averaging among neighboring cells comprising the tissue.

Altogether, these findings demonstrate the value of scnRNA-seq for quantifying disease-related changes in cell-type abundances, characterizing cellular heterogeneity, and nominating aberrant pathways in disease pathology that are restricted to sparse cellular species. In contrast to scnRNA-seq, it is unsurprising that bulk RNA-seq is insufficient for detecting transcriptional changes associated with rare cellular species; however, this does not discount the validity of the assay since the tissue-level resolution is critical for detecting the most dominant pathways in neurodegenerative disease, as discussed below.

### Synaptic Dysfunction may Contribute to DaN Susceptibility in PD

Beyond the loss of DaNs in the SNpc, intracytoplasmic inclusions known as Lewy bodies are a hallmark of PD pathology [40]. Αlpha-synuclein is the major protein component of Lewy bodies and forms aggregates in a prion-like manner in the disease state [41]. In PD, α-synuclein aggregates are associated with compromised neurotransmission, correlated with greater cognitive decline, and are believed to be a primary cause of DaN degeneration [40, 42–44]. In a normal physiological state, α-synuclein regulates neurotransmitter release, synaptic function, and plasticity, making the synapse a critical area in PD [44]. Furthermore, synaptic dysfunction related to α-synuclein aggregates may lead to PD [40]. Synaptic dysfunction in PD comprises a valuable case study for demonstrating the compatibility of bulk and scnRNA-seq, whereby transcriptional analyses at a bulk-tissue resolution are sufficient to detect the dominant pathway and define important avenues for further investigation, while scnRNA-seq can inform the cell types likely responsible for the signals observed at a bulk resolution.

Two bulk RNA-seq analyses of post-mortem midbrain tissue [45, 46] have reported changes in pathways associated with synaptic dysfunction in PD. In turn, scnRNA-seq has made multiple important contributions that corroborate the findings from bulk tissue. First, scRNA-seq analysis of LCM DaNs from the SNpc and VTA from non-PD controls revealed upregulation of pathways promoting synapse integrity in the VTA [47], suggesting that the maintenance of synaptic function is integral to the elevated resistance of DaNs in the VTA compared to DaNs in the SNpc. Second, an analysis of oligodendrocytes from the PD midbrain identified differentially expressed genes (DEG) in synaptic transmission pathways compared to non-PD controls, which coincided with a reduced fraction of myelinating oligodendrocytes in PD samples [36]. Third, an oligodendrocyte subtype, whose transcripts were enriched for processes linked to synapse organization, was found to be depleted in PD samples compared to non-PD controls [48]. Indeed, oligodendrocytes are critical in the maintenance of synaptic function in a healthy state [49], yet the snRNA-seq data suggest that a subset of oligodendrocytes lose their preservative functions in PD pathology. Interestingly, snRNA-seq revealed that oligodendrocyte and oligodendrocyte precursor cells from PD samples expressed genes that were significantly enriched within PD GWAS risk loci [50]; thus, it is possible that these genetic risk factors pose inherent risks that influence the actions of oligodendrocytes in PD pathology. It must be noted, however, that these GWAS enrichment findings derive from a pre-print manuscript which has not yet been peer-reviewed and is subject to changes following potential updates to the analyses. Nonetheless, upon amalgamating the RNA-seq evidence for synaptic dysfunction in the PD brain, it is tempting to speculate that oligodendrocyte dynamics may contribute to the elevated susceptibility of DaNs located in the SNpc. Yet, it remains to be elucidated whether a mere depletion of oligodendrocytes, a transition towards a pathogenic state, or a combination of both possibly contribute to the loss of DaNs in the PD brain.

In the prefrontal cortex, bulk RNA-seq analyses identified transcriptomic signatures related to synaptic processes in PD samples; however, these findings were attenuated upon correction for cellularity [51]. Nonetheless, snRNA-seq revealed that neuronal cells in the PD prefrontal cortex may undergo compensatory synaptogenesis as the most significantly upregulated pathways were associated with synaptic assembly [52]. While, these observations were unique to a pre-print manuscript that has not undergone peer review, the findings at a bulk resolution provide support to the conclusions. Furthermore, expressional changes related to postsynaptic pathways in excitatory neurons showed the strongest correlation with Lewy pathology score—measured by phospho-S129-α-synuclein immunohistochemistry [52]. These findings reflect the idea that alterations to the neuronal architecture in the prefrontal cortex and the accumulation of α-synuclein aggregates are associated with disrupted synaptic processes in PD pathogenesis [40]. Indeed, the convergence on synaptic-related pathways by both technologies supports its pathological role in the PD prefrontal cortex but raises a limitation with applying cell-type corrections to bulk RNA-seq data as it may discard relevant biological signals. Accordingly, the importance of validating the findings from bulk RNA-seq with the improved resolution of scnRNA-seq emerges, especially when attempting to conduct analytical procedures that address the cellularity of the transcriptional profiles.

### Glial Cells Activate Inflammatory Pathways in PD

Although neuroinflammation in the pathophysiology of PD has garnered support through epidemiological and genetic studies, many questions concerning its role in the PD disease process remain unsolved [53]. Among them is what cell types contribute to the innate immune response [53], which is primed for investigation by scnRNA-seq assays given the single-cell resolution. Further, a major limitation of bulk RNA-seq assays for investigating neuroinflammation in neurodegenerative disease is establishing whether altered expression derives from changes in cell-type proportion, regulatory events that affect gene expression, or a combination of both. While cell-type deconvolution tools may help elucidate this ambiguity, issues surrounding the accuracy of current deconvolution algorithms have been raised [54]; thus, these findings often warrant further validation by different modalities, including scnRNA-seq, which can be used to quantify both changes in cellular composition and gene expression between phenotypes.

Bulk RNA-seq analyses of the midbrain [45], prefrontal cortex [51], and frontal cortex [55] have all identified transcriptional evidence for inflammatory pathways in PD samples. The strongest signal was derived from the midbrain, as bulk RNA-seq disclosed that approximately half of the DEGs between PD samples and non-PD controls were related to the immune-inflammatory response [45]. In this analysis, cell-type deconvolution revealed equal distributions of cellular species according to disease status, suggesting that the immune-inflammatory signal resulted from regulatory events affecting gene expression, as opposed to changes in the number of cells expressing the genes. In contrast, an activated inflammatory response in the PD prefrontal cortex was inferred by bulk RNA-seq through significant increases in microglia and oligodendrocyte marker gene profiles compared to non-PD controls, without any differential gene expression to suggest an elevated inflammatory response in PD [51]. In the frontal cortex, Cappelletti et al. leveraged bulk RNA-seq data from 84 samples, allowing them to maintain power upon stratifying the PD samples into three groups according to their degree of Lewy body pathology—based on the Braak Lewy body stage—which was used as a proxy for disease progression [55]. In doing so, the authors observed that immune response pathways were upregulated at early disease stages and downregulated at the most advanced stage [55]. These data suggest that neuroinflammation in the frontal cortex may proceed the formation of Lewy bodies and play a critical role in the earliest stages of disease pathogenesis. Importantly, the stratification of PD samples by Cappelletti et al. exemplifies an important application of bulk RNA-seq, whereby the assay is not only valuable during early-stage analyses for defining the most dominant pathways in disease pathogenesis but may also be used to uncover clinico-pathological associations owing to the relative affordability of the sequencing technology and consequent larger sample sizes that facilitate patient stratification.

The findings of bulk RNA-seq regarding neuroinflammatory pathways in the Parkinsonian brain are corroborated by snRNA-seq. snRNA-seq studies on the prefrontal cortex [52], SN [48, 56, 57], and midbrain [36] also found evidence of an increased inflammatory response in PD, highlighting the role of neuroinflammation in specific cell types. In the prefrontal cortex, snRNA-seq found a higher proportion of microglia and brain resident T cells in PD samples compared to non-PD controls [52], suggesting a neuroinflammatory response and supporting the changes in cell-type proportions observed through bulk RNA-seq of this brain region. In both the midbrain and SN, an increase in the proportion of glial cells was observed in PD samples compared to non-PD controls, in accordance with recent evidence showing that neurodegenerative cues can drive the proliferation of select glial populations [57]. Glial cells from the PD midbrain showed enrichment for microglia in an activated state and astrocytes at the end of astrogliosis [36], while microglia from the SN showed transcriptional evidence for the cytokine-mediated signaling pathway that strengthened with advancing PD pathogenesis [56]. In fact, an independent snRNA-seq analysis identified a transcriptionally distinct microglia subtype overrepresented in the PD SN that showed differential expression of *ST6GAL1*, which has been shown to promote sustained signaling through the NFκB and JAK/STAT inflammatory pathways [48, 58]. Taken together, these snRNA-seq analyses of the PD midbrain support both the increase in immune cells and upregulation of immune responses uncovered by the differential gene expression signals associated with neuroinflammation that were observed at a bulk resolution.

The convergence of both sequencing technologies substantiates the validity of neuroinflammation in PD brain tissues. Importantly, bulk and snRNA-seq showed concordance regarding the basis for changes in gene expression related to inflammatory pathways in PD. For instance, in the prefrontal cortex, both assays attributed inflammatory signals to elevated proportions of microglia [51, 52], while in the midbrain, the inflammatory response was primarily attributed to changes in microglial expression profiles [36, 45]. Indeed, the neuroinflammatory results demonstrate the benefits of integrating the assay types as their complementarity and concordance provides intersectional support for the biological basis of transcriptional signals. Nonetheless, it remains unclear whether the inflammatory response in glial populations results from the PD disease trajectory, if they are causal in pathological processes, or both. Given the temporal constraints of performing RNA-seq analyses on human tissue, it is unlikely that these assays alone will sufficiently address this query.

### Mitochondrial Dysfunction Contributes to DaN Vulnerability to Degeneration

Substantial evidence has implicated mitochondrial dysfunction in the pathogenesis of PD. Mitochondrial dysfunction in PD is caused by bioenergetic defects, mutations to nuclear and mitochondrial DNA, and changes to mitochondrial dynamics, morphology, trafficking and transport, movement, and transcription [59]. Mitochondrial dysfunction coincides with oxidative stress, which is believed to cause dysfunction and misassembly of Complex 1—a key component of the oxidative phosphorylation pathway for ATP production [60]. In turn, it is believed that defects in Complex 1 contribute to the loss of DaNs in PD due to increased sensitivity of DaNs to neurotoxins [61].

Beyond the mere presence of mitochondrial dysfunction in the PD brain, an understanding of which cell types are most susceptible to this pathological feature and the molecular basis for this susceptibility requires further elucidation. Both RNA-seq assays have provided important evidence for these queries. Bulk RNA-seq has identified changes in gene expression associated with mitochondrial function in the midbrain [45], SNpc [47], and frontal cortex [55], nominating transcriptional changes associated with this pathological feature as an important component to the PD disease process. In turn, scRNA-seq identified upregulated genes involved in regulation of mitochondrial stability in DaNs of the VTA compared to those of the SNpc [47], implying that mitochondrial dysfunction may contribute to the disparity in DaN degeneration between these brain regions. Separate scnRNA-seq evidence corroborates the association between mitochondrial dysfunction and DaN vulnerability. First, scRNA-seq analyses of LCM DaNs from the SN identified DEGs associated with mitochondrial function in PD, including the upregulation of *PNMT*, which inhibits mitochondrial respiration, and can induce neurotoxin-mediated death in this cell type [62–65]. Additionally, snRNA-seq analysis found that a transcriptionally distinct SNpc DaN subtype most vulnerable to degeneration in PD displayed the strongest upregulation of NR2F2 targets [33]. NR2F2 is a transcription factor that, when overexpressed, can lead to mitochondrial dysfunction [66]. Again, we observed that the refined resolution of scnRNA-seq compliments the findings of bulk RNA-seq by singling out the cell type that may suffer the highest burden of mitochondrial dysfunction—that is, DaNs located in the ventral tier of the SNpc.

Despite the complementarity of the findings discussed above, it is unlikely that DaNs were solely responsible for the mitochondria-related signal observed in midbrain tissue at a bulk resolution given the relative sparsity of this cell type. In accordance with this hypothesis, snRNA-seq analyses of the SN observed differential expression related to mitochondrial function in PD DaNs, GABAergic neurons, and oligodendrocytes [48]. Oligodendrocytes maintain neurocircuitry by providing trophic and metabolic axonal support [67], while GABAergic neurons undergo crucial interactions with DaNs from the SNpc to regulate their firing pattern [68]. Accordingly, mitochondrial dysfunction in multiple cell types of the PD brain could lead to altered network function leading to DaN degeneration and death. The sum of RNA-seq evidence suggests that, while DaNs may be most vulnerable to the consequences of mitochondrial dysfunction, the pathological feature is not restricted to this cell type.

Importantly, the expression of mitochondrial genes is a widely used proxy for measuring RNA contamination in scnRNA-seq assays. Although computational measures can be taken to control for contamination rates, in the absence of further validation at the RNA or protein level, the convergence of multiple RNA-seq studies on common mitochondrial-related signals is necessary to ensure that the findings do not result from artifacts within the data.

### Unfolded Protein Response is Impaired in Multiple Brain Cell Types in PD Brain

The accumulation of unfolded proteins is a hallmark of neurodegeneration. Neuronal cells are particularly sensitive to protein misfolding, and prolonged periods of cell stress could trigger apoptosis [69]. To mitigate the consequences of misfolded proteins, cells activate a mitochondrial UPR, elevating the endoplasmic reticulum’s chaperone capacity for protein folding [70, 71]. Indeed, post-mortem brain samples from PD patients have shown UPR activation [72]; however, which cellular species undergo a UPR and how it may disproportionally instigate the degeneration of particular cell types remain to be fully elucidated.

Both bulk and scnRNA-seq have found evidence for the UPR in the Parkinsonian brain, with the latter technology informing the cellular species likely responsible for the signals at a bulk resolution. Specifically, bulk RNA-seq analysis of the SN found that chaperone-mediated protein folding was among the most significantly enriched pathways in PD samples [46], nominating transcriptional changes associated with this pathological feature as a major component to PD. snRNA-seq analyses of post-mortem SN [48, 56, 57] and midbrain [36] tissue corroborated the findings from bulk RNA-seq, revealing that genes involved in the UPR were differentially expressed across both neuronal and non-neuronal cell types. Specifically, snRNA-seq identified one transcriptionally distinct microglia subtype that was depleted in PD SN samples and enriched for genes involved in the UPR compared to non-PD controls [57], indicating that the microglia-mediated response against the accumulation of misfolded proteins may be impaired in PD. The analysis is currently only available as a pre-print and has not been peer-reviewed; however, the convergence of multiple analyses on the UPR in PD provides a certain degree of support to their findings. SnRNA-seq also identified a distinct astrocyte sub-type predominately seen in PD SN samples that showed unique transcriptional profiles associated with the UPR [48], suggesting that this may be a reactive astrocytic state specific to PD, which reflects a previous proposition that the UPR may generate reactive astrocytes that are fatal for neurons [73]. Regarding neurons, snRNA-seq found transcriptional enrichment of genes associated with the UPR only in DaNs and GABAergic neurons, which were significantly depleted in the SN of PD samples compared to non-PD controls, and were absent from neuronal subtypes resistant to degeneration in PD [48]. Collectively, the single-nucleus transcriptional data suggest that the UPR appears to be altered across most cell types in PD and that the selective loss of neuronal subtypes—that is, GABAergic neurons and DaNs—may reflect the sensitivity of these cells to protein misfolding. The evidence also implies that dysfunctional UPR in glial cells could impair their ability to provide support for neural health. The identification of the UPR in transcriptionally distinct glial and neuronal cell types in PD is a valuable illustration of the power of scnRNA-seq for unravelling cell-type specific pathway perturbations and the corresponding pathological consequences in neurodegenerative disease.

A similar relationship regarding the complementarity of bulk and snRNA-seq evidence for the UPR was observed in the PD prefrontal cortex. Specifically, bulk RNA-seq found that pathways related to the endoplasmic reticulum and the UPR emerged as the top differential gene expression signatures in PD after correction for cell-type composition in the bulk tissue [51]. At a single-nucleus resolution, Zhu et al. observed that the degree of Lewy pathology across PD samples was inversely correlated with chaperone expression in excitatory neurons, and that PD samples showed downregulated UPR-associated DEGs compared to non-PD controls [52]. These data suggest that reductions in chaperone activity may propel α-synuclein aggregation in this brain region. The study by Zhu et al. provides a valuable illustration of scnRNA-seq for elucidating clinico-pathological associations. However, given the relatively small sample size (six PD; six non-PD controls), these findings warrant further validation from a cohort-scale analysis. Certainly, the relative affordability of bulk RNA-seq nominates this technology as a valuable tool to obtain supporting evidence with a larger sample size.

### Contrasting Applications of Bulk and scnRNA-seq Technologies in Neurodegenerative Disease

The choice between bulk and scnRNA-seq technologies for studying neurodegeneration depends on the underlying aims of the research. While bulk RNA-seq can identify overarching transcriptional differences between conditions by uncovering DEGs that are abundant enough to generate signals in whole tissues, scnRNA-seq provides insight into the transcriptional landscape of individual cells and the cellular heterogeneity comprising the tissue of interest. However, each technology has limitations that restrict utility for unravelling the pathology of multifactorial neurodegenerative diseases.

The cost of transcriptomic investigations to reach a desired level of power is of practical importance. Currently, the cost of scnRNA-seq is still prohibitive for studies involving hundreds of thousands of cells. We assembled a generic protocol and price comparison between the sequencing technologies and estimated costs of $55–320 and $350–700 per sample for bulk RNA-seq and scnRNA-seq, respectively (2.19–6.36 × for scnRNA-seq; Table 2). However, prior estimates have stated that the cost of scnRNA-seq may reach 30-fold greater than bulk RNA-seq [54]. Furthermore, while automated library preparation methods using programmable machines can facilitate bulk RNA-seq, this technology does not yet exist for scnRNA-seq and, therefore, transcriptional profiles produced at a single-cell resolution currently require entirely manual library preparation. Thus, scnRNA-seq workflows are more laborious, time-consuming, and require specialized equipment for single-cell isolation, limiting the feasibility of cohort-scale analyses. The sample sizes of the RNA-seq studies reviewed here reflect the disparity in per sample cost and labor requirements between tissue-level and single-cell transcriptomics; sample sizes (combining case and control) ranged from 20 to 84 (mean = 49; median = 42 in 6 studies) subjects for bulk RNA-seq analyses and 6 to 33 (mean = 17; median = 15 in 10 studies) subjects for scnRNA-seq analyses (Table 1).

**Table 2:** Protocol and price comparison for bulk and single-cell/single-nucleus RNA sequencing analysis

Bulk RNA-seq		scnRNA-seq	
**RNA isolation kit**	$5–20 per sample	**GEM creation (10X)**	$300–600 per set of beads (2000–10000 cells)
**Library preparation for sequencing**	$50–300 per sample	**Library preparation for sequencing**	$50–100
**Total cost per sample**	$55–320	**Total cost per sample/2000–5000 cells**	$350–700
**Sequencing depth per sample**	50–100 M reads	**Sequencing depth per cell**	30–100 K reads
**Amount of sequencing required for differential gene expression experiment**	600 M reads	**Amount of sequencing required for differential gene expression experiment**	750 M reads for six samples at 50 K reads per cell, assuming 5000 cells per sample

For bulk RNA sequencing (RNA-seq), in general, an RNA isolation kit is required to isolate RNA from whole tissue, followed by library preparation for sequencing. For single-cell or single-nucleus RNA-seq (scnRNA-seq), assuming droplet-based sequencing, a gel bead-in-emulsion (GEM) creation step is required to partition single cells or nuclei, followed by library preparation for sequencing. Prices are shown in Canadian dollar values

Small cohorts are a major limitation in scnRNA-seq studies, especially for neurodegenerative diseases, such as PD, that show heterogeneous presentations including variable age of onset, symptoms, and progression rates [74]. Not only do small sample sizes fail to recapitulate the etiological diversity of these diseases, but they also prevent the stratification of samples to detect covariate-related differences such as age, sex, and RNA integrity number. Further, small sample sizes may lack sufficient power to detect associations between transcriptional changes and clinical or pathological features such as age at onset, survival, and Lewy body pathology. Certainly, the use of scnRNA-seq for detecting clinco-pathological associations with small sample sizes is not impossible. For example, Zhu et al. observed that Lewy pathology was inversely correlated with chaperone expression in excitatory neurons of PD samples. However, the relatively small sample size of 12 individuals warrants skepticism that can only be mitigated by replication at a cohort scale [52]. Ultimately, the current cost of single-cell technologies often restricts the rigorous, comprehensive characterization of transcriptional changes in neurodegenerative diseases despite its unprecedented resolution.

In contrast to scnRNA-seq, the relative affordability of bulk RNA-seq makes its application to large sample sizes feasible, rendering it an exceptional tool for establishing clinico-pathological associations. The study from Cappelletti et al. provides a notable example of this application. Harnessing 84 subjects (61 PD; 23 non-PD controls) allowed the authors to stratify their PD samples according to their degree of Lewy body pathology [55]. In doing so, they revealed important transcriptional discrepancies between PD samples and inferred temporal fluctuations in inflammatory pathways throughout PD pathogenesis. Maintaining sufficient statistical power to establish these associations after sample stratification requires cohort-scale analyses, and, at present, the feasibility of bulk RNA-seq is unparalleled for these applications.

Another factor that favors the use of bulk RNA-seq over scnRNA-seq assays is the maturity and consensus of the procedures for data analysis. The analytical best practices for bulk RNA-seq data, including transcript identification, transcript quantification, and differential gene expression, have been well established [75]. In contrast, the analytical processes for scnRNA-seq, including methods for data filtering, integration, and cell-type annotation, are all in development. Notably, how to calculate differential gene expression between groups is controversial. Currently, “pseudo-bulk” analysis—whereby the expression values of a group of cells from the same individual are aggregated prior to differential expression analysis and treated as biological replicates—is considered the best practice [76]. However, the pseudo-bulk method is not usually applied, and individual cells from all subjects within a group are considered as biological replicates.

Nonetheless, a major limitation of bulk RNA-seq is confounding due to the differences in cellular composition between samples, making it difficult to determine whether transcriptional changes result from regulatory events that affect the expression of a gene, a change in the number of cells expressing the gene, or both. Although differences in cell-type composition may represent valid disease-related changes, they may also stem from technical variability such as differences in the proportions of cellular species captured during tissue extraction. Regardless of the underlying cause, differences in cell-type composition can dramatically influence the gene expression profiles and bias downstream analyses. The magnitude of this limitation was emphasized in Nido et al.’s bulk RNA-seq analysis of the PD prefrontal cortex, where they observed that the main axis of variation in gene expression was significantly correlated with RNA quality and cell-type composition [51]. Although understanding the changes in gene expression due to disease-related alterations in cell-type abundance pose significant implications for unravelling neurodegenerative disease pathogenesis, bulk RNA-seq alone is insufficient to confidently resolve this ambiguity.

One method to compensate for the confounding effects of differences in proportions of cell types in bulk RNA-seq data is through computational deconvolution algorithms. These methods infer cell-type abundances obtained from bulk RNA-seq profiles by harnessing either scnRNA-seq data from the same tissue [77–80] or cell-type specific gene expression signatures [81–83]. Yet, concerns about the performance of deconvolution methods have been raised [54, 81, 84], and their accuracy depends on the suitability of the reference data, including the cellular composition of the expression matrix, the methods employed for data transformation and normalization, and the clustering resolution used to identify distinct cellular species [54]. In accordance with this controversy, we observed instances of both agreement and disagreement between deconvolution algorithms applied to bulk RNA-seq data and snRNA-seq. Both bulk [51] and snRNA-seq [52] identified a higher proportion of microglia in the PD prefrontal cortex compared to non-PD controls, but bulk RNA-seq of the midbrain failed to detect a difference in the abundance of glial cells in PD samples compared to non-PD controls [45], whereas snRNA-seq of this brain region emphasized notable changes in cellularity according to disease status [36]. We also observed that correcting bulk RNA-seq data for cellularity may attenuate relevant biological signals. For example, bulk RNA-seq identified transcriptomic signatures in the PD prefrontal cortex associated with synaptic processes that were attenuated upon correction for cellularity [51]. However, snRNA-seq revealed that the most significantly upregulated pathways in neuronal cells from the PD prefrontal cortex were associated with synaptic processes [52]. Thus, even though deconvolution methods for bulk RNA-seq data continue to improve, the validity of their predictions is only substantiated when supported by different modalities and, at present, does not represent an adequate substitute for scnRNA-seq.

Differences in read abundance, sequencing depth, and mRNA capture are also important factors when considering bulk and scRNA-seq assays. scnRNA-seq assays face issues with “drop-out” events, whereby there is a probability that an expressed gene will not be detected by scnRNA-seq methods. The drop-out rate for modern scnRNA-seq assays remains unclear, but estimates range between 60 and 90% [85]. It also remains to be elucidated whether drop-out events are random or mRNA transcript selective; transcripts of certain lengths, locations, or stage in RNA processing might be better captured. Another limitation of scRNA-seq is that it does not capture RNA from outside of the cytoplasm. This is particularly relevant for studying neurons in neurodegenerative disease given that RNA in the cellular processes, such as axons and dendrites, will be lost as they are removed during dissociation. In contrast, if RNA is present in a cell, it will be captured by bulk RNA-seq. Additionally, dissociation and droplet formation in scnRNA-seq workflows appear to select against neurons and therefore mis-represent the cell populations in the brain. A notable example of this came from Smajic et al.’s snRNA-seq analysis of the midbrain, whereby DaNs comprised only 0.18% of the cells in their experiment [36]; however, 15 to 20% of cells in the SN are DaNs, which were lost during cell capture.

Sequence alignment is also less favourable in scnRNA-seq assays compared to bulk RNA-seq because only a small portion of the 3′ or 5′ mRNA sequences are sequenced. Specifically, for 10X Genomics technologies, an alignment of 65% is considered acceptable. In bulk RNA-seq, the entire mRNA sequence is sequenced, resulting in alignments > 99.9% and facilitating the prediction of splice variants and detection of single nucleotide polymorphisms across the whole transcript. Thus, bulk RNA-seq affords more in-depth transcriptional information despite a lack of cell-specific data.

### Maximizing the Potential of RNA-seq in Future Applications to Neurodegenerative Disease

Bulk and scnRNA-seq provide critical insights into the pathogenesis of neurodegenerative disease. Throughout our review of the PD RNA-seq literature, we observed that the evidence from bulk and scnRNA-seq intersected at the most frequently perturbed pathways in PD, including synaptic dysfunction, neuroinflammation, mitochondrial dysfunction, and the UPR. Beyond the observed concordance between sequencing technologies, scnRNA-seq frequently elucidated the cellular species that were likely driving the changes in gene expression observed in bulk tissue, including microglia and the immune-inflammatory response in PD midbrains and non-neuronal cell types and the UPR in the PD SN. Furthermore, integration across sequencing technologies allowed us to infer which changes in gene expression observed at a bulk resolution likely resulted from underlying changes in cell-type proportions between conditions and which were likely the result of disease-related regulatory events that led to differential gene expression. One example includes the enrichment of microglia in an activated state in the PD midbrain identified by snRNA-seq [36], and changes in gene expression associated with the immune-inflammatory response in this same brain region at a bulk resolution [45].

Undoubtedly, the high resolution of scnRNA-seq has revolutionized the molecular understanding of neurodegenerative disease through its ability to characterize transcriptional changes at the resolution of a single cell. However, because of the technical and financial challenges associated scnRNA-seq, bulk RNA-seq is likely to remain the most frequently used assay for the foreseeable future. Given that the findings of bulk RNA-seq remain robust when compared to those achieved at a single-cell resolution, coupled with its suitability for large-scale clinico-pathological association studies, it is apparent that modern single-cell technologies are far from rendering bulk RNA-seq obsolete for analysis of brain tissue. In fact, we conclude that the continued application of both assays will provide the greatest insight into neurodegenerative disease pathology utilizing both cell-centered and whole tissue-level contexts.

Moving forward, the direct integration of bulk and scnRNA-seq data may prove to yield the greatest rate of discovery in neurodegenerative disease biology as the limitations of each method are compensated for by the strengths of the other. This methodology was recently outlined in a study of Alzheimer’s disease, where researchers harnessed snRNA-seq from a few samples to generate a high-resolution cellular map of the frontal cortex, in order to infer the cellular architecture of a cohort scale, clinically characterized bulk RNA-seq dataset [86]. Thus, the researchers obtained cellular-level data at a sample size necessary for identifying clinico-pathological associations. Similar approaches have also been employed in cancer research, where computational tools such as EcoTyper [87] aim to unravel distinct cellular states and communities in bulk RNA-seq data based on scnRNA-seq reference expression profiles. We reason that these tools should be applied across neurodegenerative diseases to inform how cellular environments interact to promote neurodegeneration, at a sample size necessary for robust statistical associations. Undoubtedly, continued efforts to maximize the accuracy of projecting scnRNA-seq data onto cohort-scale bulk RNA-seq data is a worthwhile endeavour in neurodegerative disease research.
